# Acute pulmonary embolism combined with acute myocardial infarction as the first manifestation of acute leukemia: a case report

**DOI:** 10.3389/fcvm.2023.1259548

**Published:** 2023-09-13

**Authors:** Shuzhan Zheng, Sha Luo, Yong Luo, Dan Liu, Wenwu Zheng, Qing Peng

**Affiliations:** ^1^ Department of Cardiology, The Affiliated Hospital of Southwest Medical University, Luzhou, China; ^2^ Department of Respiratory and Critical Care Medicine, The Affiliated Hospital of Southwest Medical University, Luzhou, China; ^3^ Inflammation & Allergic Diseases Research Unit, The Affiliated Hospital of Southwest Medical University, Luzhou, China

**Keywords:** acute myocardial infarction, pulmonary embolism, acute myeloid leukemia, thrombocytopenia, percutaneous coronary intervention

## Abstract

Thrombotic complications in acute myeloid leukemia (AML) are uncommon due to coagulation dysfunction and thrombocytopenia. We report a unique case of AML presenting as concomitant pulmonary embolism and atypical acute myocardial infarction. A 67-year-old male experienced persistent bilateral chest pain. Despite an unremarkable electrocardiogram, elevated D-dimer and mildly increased troponin T levels prompted further investigation, leading to the diagnosis of simultaneous pulmonary embolism and acute myocardial infarction. The patient underwent percutaneous coronary intervention and received triple antithrombotic therapy. However, antithrombotic therapy was discontinued following a sharp decline in hemoglobin and platelet counts, and the patient subsequently developed persistent fever. AML was diagnosed via bone marrow biopsy. Chemotherapy was not initiated due to the patient's deteriorating condition, and he ultimately succumbed to presumed intracranial bleeding.

## Introduction

Thrombotic complications in acute myeloid leukemia (AML) are rare due to coagulopathy and thrombocytopenia. Paradoxically, thrombocytopenia and thrombosis can coexist, leading to treatment dilemmas, as thrombosis may be fatal and become the primary cause of death. Additionally, concomitant arterial and venous embolisms are seldom reported in association with AML. This case is notable for the simultaneous occurrence of acute pulmonary embolism (PE) and acute myocardial infarction (AMI) as the initial manifestation of AML, which was treated with antithrombotic therapy until the final diagnosis of acute leukemia. Atypical clinical presentations of acute myocardial infarction may signify early-stage AML.

## Case

A 67-year-old male patient was admitted to the hospital with a complaint of bilateral chest pain and weakness lasting for five days. The patient reported that the pain was persistent, albeit tolerable, and not associated with exercise or breath. He denied experiencing symptoms such as cough, dyspnea, diaphoresis, or lower limb edema. His pertinent medical history included a well-controlled hypertension for three years and a recent weight loss of 10 kg over the past year. The patient was a non-smoker and reported infrequent alcohol consumption.

The patient's vital signs upon admission were within normal limits, with a blood pressure of 115/72 mmHg, a heart rate of 80 bpm, a respiratory rate of 18 bpm, and an oxygen saturation (SpO2) of 98% while breathing room air. Physical examination revealed scattered moist rales across the bilateral lower lung fields. Cardiac and abdominal examinations were unremarkable, with no evidence of edema in the lower extremities.

The results of the complete blood count (CBC) revealed a white blood cell count of 8.51 × 10^9^/L, a hemoglobin level of 114 g/L, and a platelet count of 96 × 10^9^/L. The coagulation panel showed a prothrombin time (PT) of 12.6 s, a D-dimer level of 94.83 ug/ml [normal range (NR) 0–0.5], a fibrinogen level of 6.53 mg/dl (NR 1.8–3.5), fibrinogen degradation products (FDP) level of 270.70 ug/ml (NR 0–5), and antithrombin III (ATIII) level of 83% (NR 80–120). The cardiac marker showed a high-sensitive troponin T level of 0.357 μg/l (NR 0–0.014). Liver and renal function tests were unremarkable, while procalcitonin levels were elevated at 0.29 ng/ml (NR 0–0.05).

**Figure 1:**
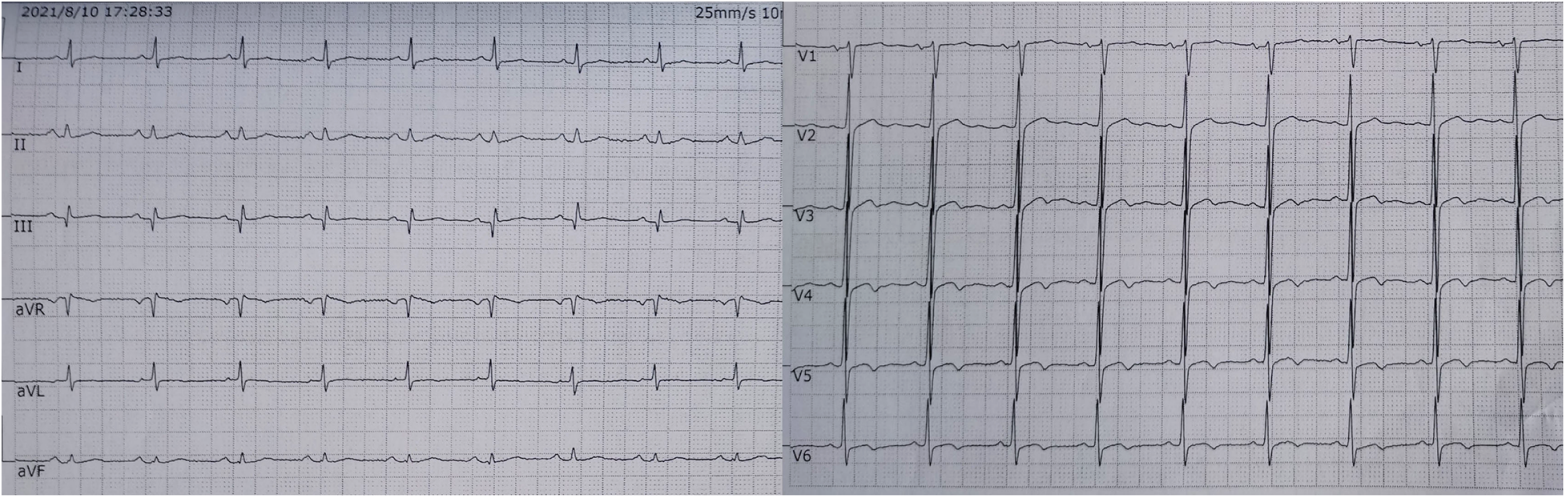
The electrocardiogram showed no ST segment changes but a Q wave in lead III.

**Figure 2:**
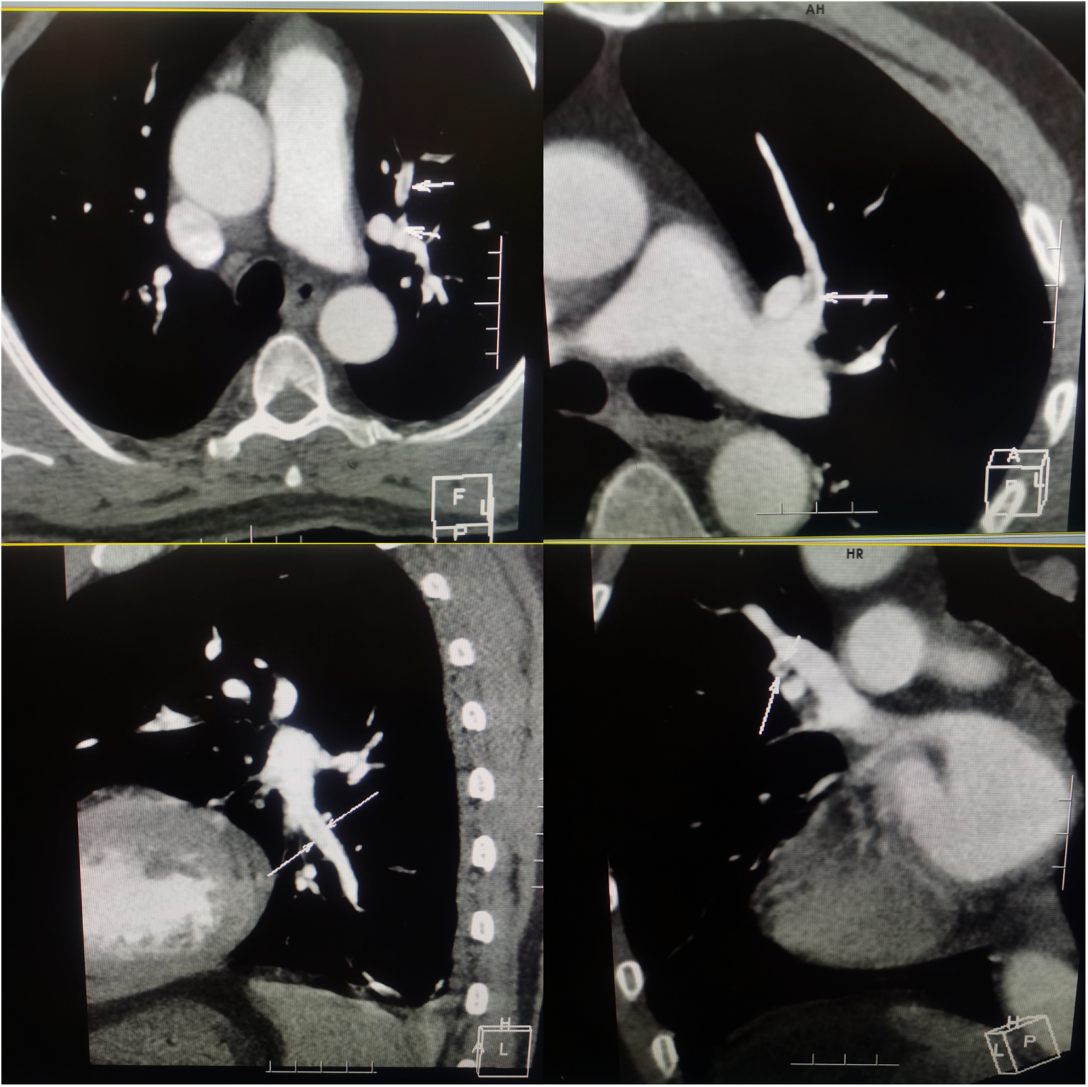
Computered tomography angiography of chest showing embolization in the left upper branches and bilateral lower pulmonary arteries.

The electrocardiogram showed no ST segment changes but a Q wave in lead III (Figure 1). The computed tomography (CT) of the chest revealed a 1.0 × 1.1 cm solid nodule in the right upper lobe of the lung, with a shallow lobulated edge, and mild to moderate homogeneous enhancement, and exudation in the lower lobe of the lungs, mild bilateral pleural effusion. CT angiography showed embolization in the left upper branches of the pulmonary arteries and bilateral lower pulmonary arteries (Figure 2). However, no thrombus was found in the lower limb veins. Pulmonary embolism was diagnosed, and the patient was given 0.6 ml enoxaparine subcutaneously every 12 h for anticoagulation.

**Figure 3:**
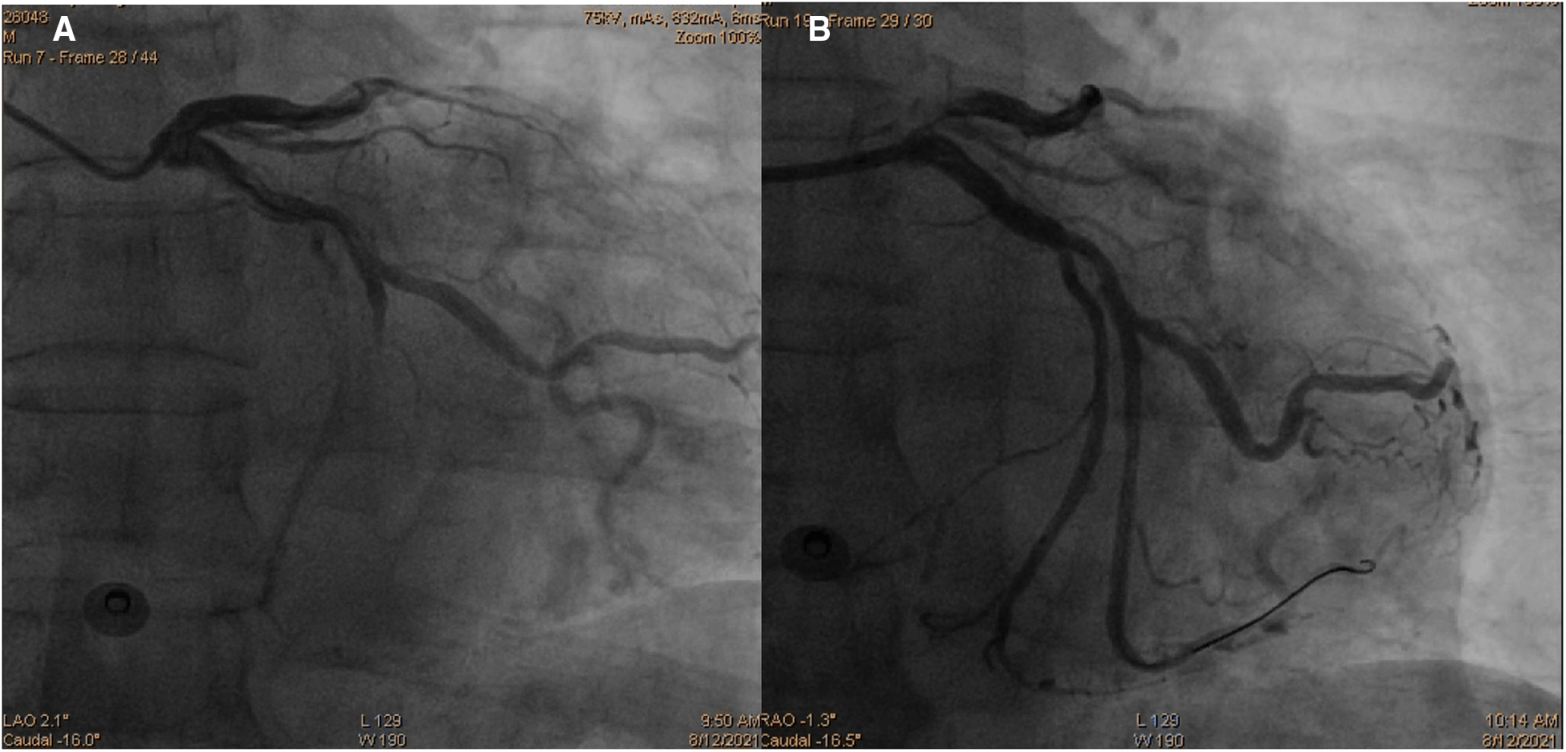
Coronary angiography images demonstrating occlusion in the middle segments of the left circumflex artery (**A**) and revascularization of the left circumflex artery after percutaneous coronary intervention (**B**).

Initially, acute coronary syndrome was not considered due to the absence of angina and no ST segment change in the electrocardiogram. However, moderate stenosis in the left coronary artery was identified by CT angiography. Subsequent coronary angiography revealed occlusion in the middle segments of the left circumflex artery (LCX), 50% stenosis in the middle segment of the left anterior descending artery, and 80% stenosis in the middle segment of the right coronary artery. A drug-eluting stent (DES) was successfully implanted in the middle segment of the LCX, achieving thrombolysis in myocardial infarction (TIMI) grade III flow (Figure 3). The patient was given 100 mg aspirin with 75 mg clopidogrel daily for antiplatelet aggregation and 20 mg atorvastatin to stabilize the plaque after the procedure. To identify the cause of concomitant thrombosis in the pulmonary arteries and coronary artery, autoantibody spectrum, anticardiolipin antibody, tumor markers of the respiratory system, rheumatoid factor, and immunoglobulin levels were tested, but no abnormalities were found.

On the second day of hospitalization, the patient's hemoglobin level decreased to 106 g/L, and platelet counts dropped to 55 × 10^9^/L. Furthermore, the D-dimer levels increased, and urinary occult blood was detected. Suspecting heparin-induced thrombocytopenia, the patient's medication was switched from enoxaparin to rivaroxaban, with a daily dose of 10 mg. Following the switch, the urinary occult blood became negative on two consecutive tests. On the third day, the patient developed a fever, and piperacillin sodium, tazobactam, and cephalotazidine moxifloxacin were administered to treat the infection. Hepatitis virus screening and autoimmune hepatitis tests, along with tests for antibodies to EB virus, cytomegalovirus, rubella virus, and herpes simplex virus, galactomannan tests, and blood bacterial cultures were all negative.

**Figure 4:**
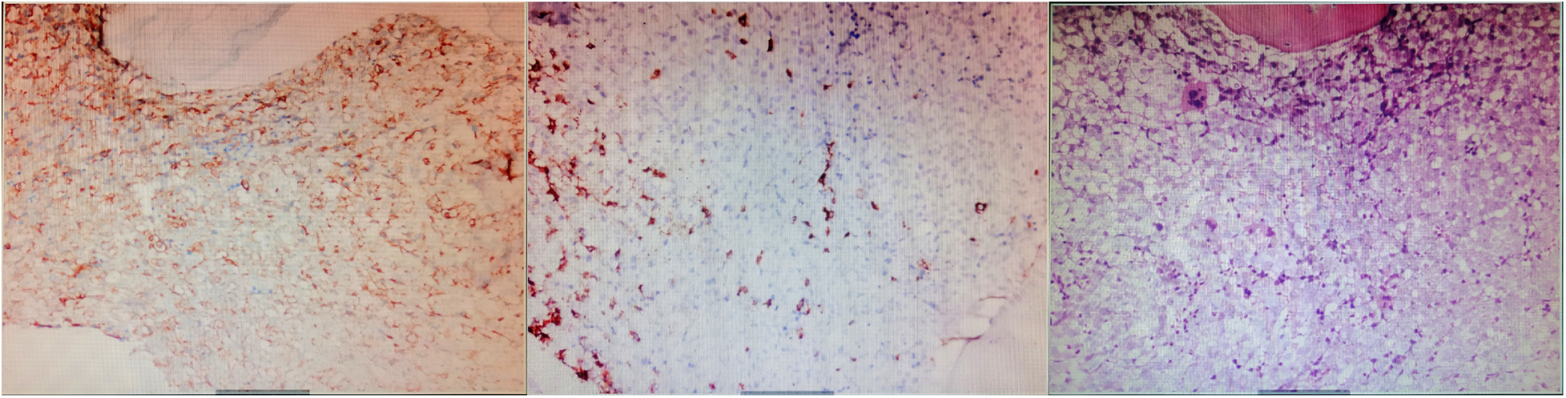
Immuohistochemical stainin of bone marrow biopsy (×200). The results showed a large amount of necrosis in the bone marrow tissue, with hyperactive proliferation of blasts in the necrotic part and few megakaryocytes and lymphocytes.

On the sixth day of hospitalization, pancytopenia with 13% blasts was detected in the patient's peripheral blood. The following day, ecchymosis was observed at the vein puncture site on the wrist, and the white blood cell counts dropped to 2.61 × 10^9^/L, with hemoglobin at 61 g/L and platelets at 8 × 10^9^/L, with 17% blasts. The coagulation panel revealed disseminated intravascular coagulation, as evidenced by a prolonged PT (22.8 s), elevated D-dimer levels (>20.00 μg/ml), and decreased fibrinogen levels (5.03 mg/dl), with FDP levels >150.00 μg/Ml and normal ATIII level (88%). The Coombs test was negative. Following consultation with a hematologist, aspirin, clopidogrel, and rivaroxaban were discontinued, and red cell and platelet transfusions were administered, resulting in platelet counts increasing to 37 × 10^9^/L and PT decreasing to 15.2 s. Fortunately, no fresh ecchymosis or massive hemorrhage occurred. A bone marrow smear revealed hyperplasia of nucleated cells with necrotic cell remnants, confirming the diagnosis of acute myeloid leukemia (AML). The bone marrow biopsy showed a large amount of necrosis in the bone marrow tissue, with hyperactive proliferation of blasts in the necrotic part and few megakaryocytes and lymphocytes (Figure 4). Immunophenotyping was not performed due to few nucleated cells. Immunohistochemical examination revealed CD117 (−), CD34 (−), MPO (granulocyte+), CD68/PG-M1 (+), CD71 (nucleated red blood cell+), CD61 (megakaryocyte+), CD3 (−), CD20 (-), CK-L (−), TTF-1 (−), NapsinA (−), CK7 (−), PSA (−), and P504s (−).

Despite the initial improvement, the patient's condition deteriorated in the following days, with persistent fever and unstable hemodynamics. The platelet count fell to 6 × 10^9^/L, and chemotherapy was not initiated due to the worsening condition. On the 16th day of admission, the patient lost consciousness, and his heart rate gradually decreased, with blood pressure eventually collapsing, leading to presumed cerebral bleeding and subsequent death.

## Discussion

Thrombosis is a rare and atypical complication of acute leukemia that may lead to misdiagnosis and improper treatment at the early stages of the disease. In this case, an elderly male patient presented with acute PE combined with AMI, received percutaneous coronary intervention (PCI) and triple antithrombotic therapy. However, the progressive decline in hemoglobin and platelet count suggested hematological disease, and the patient was ultimately diagnosed with AML. This case is relatively rare clinically since anemia and bleeding are common initial presentations, as normal bone marrow hematopoietic function is inhibited by leukemic cell proliferation and thrombocytopenia. In the literature, the association of venous thromboembolic events with hematological malignancies is well documented (1). However, limited data is available for arterial thromboembolism (ATE), ATE like stroke, acute myocardial infarction, or lower extremity arterial embolism (2, 3), and arteriovenous thrombosis (3) have been sparsely reported in leukemia.

This patient had a rapid onset and short course of chest pain, with no risk factors for PE before. When PE and AMI coexist in the setting of thrombocytopenia, it suggests that the nature of arteriovenous thrombosis is driven by underlying pathology rather than seeming diseases. Althouth the mechanism of thrombosis in acute leukemia is not yet clear, several pathogenic mechanisms have been proposed. Leukemic blasts express procoagulant proteins (such as tissue factor, TF) which can induce activation of the coagulation cascade and platelet aggregation. In addition, inflammatory cytokines (tumor necrosis factor-*α* and interleukin-1b) and microparticles secreted by leukemic cells stimulate the expression of the prothrombotic phenotype of platelets and leukocytes (4). Meanwhile, they promote the upregulation of TF and plasminogen activator inhibitor type 1 expression and the downregulation of thrombomodulin in endothelial cells (5). Besides, leukocytosis and leukostasis resulting from high blast cell count increased blood viscosity which impede blood flow (6). Moreover, it's worth noting that chemotherapy further promotes thrombus formation which is related to blasts'death and release of procoagulant factors in the circulation (7). Other reasons related to thrombosis include venous catheter procedures or infections (8). It has been reported that anticoagulant factor protein C, protein S, and ATIII decreased in varying degrees in leukemia patients (9). In this patient, the leukocyte count was normal with no blasts in peripheral blood at admission. The patient neither received chemotherapy nor underwent any invasive procedure before. Antiphospholipid antibody syndrome was excluded by negative anticardiolipin antibody test. Hypercoagulable and inflammatory state caused by blasts and pulmonary infection, combined with coexist atherosclerosis lesion might promote the simultaneous PE and AMI. Besides, although no change of ATIII level was observed, it's a limitation that other anticoagulant factors were not detected in this case, it was speculated that some anticoagulation defects might have given rise to thrombosis in leukemia.

Concomitant AMI and PE are rare and often require double antiplatelet (DAPT) and anticoagulant therapy to reduce new thrombotic events. Unfractionated heparin or low molecular weight heparin (LMWH) is recommended in the acute phase to rapidly extinguish thrombin and fibrin clot generation, which further prevents thrombus extension. Direct oral anticoagulants (DOACs) have been shown to be as effective as conventional treatment for venous thromboembolism (10). However, in the setting of leukemia with thrombocytopenia, triple antithrombotic therapy is worth exploring, as leukemia-related thrombocytopenia, platelet dysfunction, and systemic coagulation abnormalities increase the risk of bleeding. Unfortunately, despite recent guidelines addressing the management of VTE in cancer patients (11), there are no unambiguous data in dealing with thrombosis in the leukemic population. LMWH is still the standard treatment for VTE in leukemia, while data for DOACs is lacking since AML patients with thrombocytopenia were often excluded in prospective randomized trials (12). For patients with thrombocytopenia, dosing of anticoagulation should be modulated according to platelet count: Full therapeutic dose with LMWH should be considered in patients with platelet count >50 × 10^9^/L. In patients with persistent, severe thrombocytopaenia (<50 × 10^9^/L), for acute VTE at high risk of recurrence (within 30 days), platelet transfusions should be considered to maintain a count >50 × 10^9^/L and to continue a full dose anticoagulation. Anticoagulation must be withheld with platelets <25 × 10^9^/L (13).

PCI and following DAPT is the standard therapy for AMI patients. However, managing concomitant bleeding risk caused by antithrombotic drugs and stent thrombosis secondary to drug withdrawal due to bleeding events is challenging for treating patients with thrombocytopenia. Although the incidence of stent thrombosis was not significantly different between the two groups in a meta-analysis of all-cause mortality of PCI patients with or without baseline thrombocytopenia, gastrointestinal bleeding, intracranial hemorrhage, and hemorrhagic stroke after PCI were significantly higher than those of patients with normal platelets (14). To reduce bleeding risk, some experts suggest using bare metal stents or drug-eluting balloons to shorten the time of DAPT (15). Besides, the type of antiplatelet drugs and time of DAPT should be adjusted according to the platelet count: If the platelet count exceeds 10,000/ µl, aspirin can be used, while adding clopidogrel when count exceeds 30,000/ µl. If the platelet count is less than 50,000/ µl, the duration of DAPT should be 2 weeks after PTCA, 4 weeks after bare metal stenting, and 6 months after the second and third generation DES implantation. However, Prasugrel, Ticagrelor, and glycoprotein IIb/IIIa antagonists are not recommended (16). According to EHA guidelines, in secondary prevention of ATE in patients with platelets between 50 and 70 × 10^9^/L, single antiplatelet therapy (SAPT) is recommended, but be withheld if platelets <25 × 10^9^/L (13). If the platelet count is lower than 20 × 10^9^/L or the patient has fever, leukocytosis, sudden decrease of platelet count or other blood coagulation disorders, prophylactic platelet transfusion is required (17). In this case, the patient received DES implantation and was treated with a standard dose of enoxaparin and DAPT without knowing the existence of leukemia. However, on the second day after PCI, enoxaparin was replaced by rivaroxaban due to platelet decline. On the fourth day after PCI, antithrombotic drugs were discontinued due to a sharp drop in hemoglobin and platelets. Platelet transfusion was given when the platelet count was 8,000/ μl for bleeding prevention. The patient did not develop massive hemorrhage despite low platelets nor stent thrombosis without antithrombotic therapy until he died of irreversible bone marrow necrosis and possible intracranial bleeding. Thus, closely observing changes in the platelet count and timely adjusting the strategy according to the platelet value is crucial. Moreover, multidisciplinary management is also important in tackling the patient's complex situation.

## Conclusion

The initial symptoms of AML may not be typical, and thrombotic complications such as acute pulmonary embolism and acute myocardial infarction are rare but can present and lead to treatment dilemmas, further increasing the mortality of AML. It is essential to further elucidate the specific mechanism of arteriovenous thrombosis in the context of thrombocytopenia. Since there is no standard therapy, the strategy should be carefully evaluated based on individual characteristics and should involve multidisciplinary management.

## Data Availability

The original contributions presented in the study are included in the article/Supplementary Material, further inquiries can be directed to the corresponding author.

## References

[1] Rashidi A Silverberg ML Conkling PR Fisher SI. Thrombosis in acute promyelocytic leukemia. Thrombo Res. (2013)131(4):281–9. 10.1016/j.thromres.2012.11.024 23266518

[2] Muñiz AE. Myocardial infarction and stroke as the presenting symptoms of acute myeloid leukemia. J Emerg Med. (2012)42(6):651–4. 10.1016/j.jemermed.2009.04.061 19500934

[3] Hashemi A Gerges F Naqvi HR Kotlar I Moscatelli S Hashemi A A rare presentation of an elderly patient with acute lymphocytic leukemia and platelet count of zero associated with ST-elevation myocardial infarction, pulmonary thromboembolism in the setting of SARS-CoV 2:a case report. Egypt Heart J. (2021)73(1):39. 10.1186/s43044-021-00162-9 33932169 PMC8088204

[4] Horowitz NA Brenner B. Thrombosis in hematological malignancies: mechanisms and implications. Thromb Res. (2020)191:S58–62. 10.1016/S0049-3848(20)30398-4 32736780

[5] Rickles FR Falanga A Montesinos P Sanz MA Brenner B Barbui T. Bleeding and thrombosis in acute leukemia: what does the future of therapy look like? Thromb Res. (2007)120(SUPPL.2):99–106. 10.1016/S0049-3848(07)70137-8 18023721

[6] Manogna D Sham R. Acute myocardial infarction as initial manifestation of acute myeloid leukemia: a rare manifestation of leukostasis. Cureus. (2020)12(8):e9551. 10.7759/cureus.9551 32775121 PMC7405964

[7] Herrmann J Yang EH Alliescu CA. Vascular toxicities of cancer therapies: the old and the new-an evolving avenue. Circulation. (2016)133(13):1272–89. 10.1161/CIRCULATIONAHA.115.018347 27022039 PMC4817363

[8] Del Principe MI Del Principe D Venditti A. Thrombosis in adult patients with acute leukemia. Curr Opin Oncol. (2017)29(6):448–54. 10.1097/CCO.0000000000000402 28841588

[9] Dixit A Kannan M Mahapatra M Choudhry VP Saxena R. Roles of protein C, protein S, and antithrombin III in acute leukemia. Am J Hematol. (2006)81(3):171–4. 10.1002/ajh.20546 16493609

[10] Streiff MB Agnelli G Connors JM Crowther M Eichinger S Lopes R Guidance for the treatment of deep vein thrombosis and pulmonary embolism. J Thromb and Thrombolys. (2016)41(1):32–67. 10.1007/s11239-015-1317-0 PMC4715858 26780738

[11] Falanga A Ay C Di Nisio M Gerotziafas G Jara-Palomares L Langer F Venous thromboembolism in cancer patients: ESMO clinical practice guideline. Ann Oncol. (2023)34(5): 452–67. 10.1016/j.annonc.2022.12.014 36638869

[12] Falanga A Gal GL Carrier M Abdel-Razeq H Ay C Martin AJM Management of cancer-associated thrombosis: unmet needs and future perspectives. TH Open. (2021)5(3):e376–86. 10.1055/s-0041-1736037 34485812 PMC8407937

[13] Falanga A Leader A Ambaglio C Bagoly Z Castaman G Elalamy I EHA Guidelines on management of antithrombotic treatments in thrombocytopenic patients with cancer. HemaSphere. (2022)6(8):e750. 10.1097/HS9.0000000000000750 35924068 PMC9281983

[14] Long MY Ye ZL Zheng J Chen WX Li L. Dual anti-platelet therapy following percutaneous coronary intervention in a population of patients with thrombocytopenia at baseline: a meta-analysis. BMC Pharmacol Toxicol. (2020)21(1):31. 10.1186/s40360-020-00409-2 32334636 PMC7183593

[15] Iliescu CA Grines CL Herrmann J Yang EH Cilingiroglu M Charitakis K SCAI Expert consensus statement: evaluation, management, and special considerations of cardio-oncology patients in the cardiac catheterization laboratory (endorsed by the cardiological society of India, and sociedad latino Americana de cardiologıa intervention). Catheter Cardio Inte. (2016)87(5): E202–23. 10.1002/ccd.26379 26756277

[16] McCarthy CP Steg G Bhatt DL. The management of antiplatelet therapy in acute coronary syndrome patients with thrombocytopenia: a clinical conundrum. Eur Heart J. (2017)38(47):3488–92. 10.1093/eurheartj/ehx531 29020292 PMC5837661

[17] Iliescu C Durand JB Kroll M. Cardiovascular interventions in thrombocytopenic cancer patients. Tex Heart Inst J. (2011)38(3):259–60. 21720465 PMC3113145

